# Effectiveness of a rehabilitation program involving functional proprioceptive stimulation for postural control and motor recovery among stroke patients: a double-blinded, randomized, controlled trial

**DOI:** 10.1186/s12984-025-01678-w

**Published:** 2025-07-04

**Authors:** Agnieszka Wiśniowska-Szurlej, Justyna Leszczak, Justyna Brożonowicz, Gabriela Ciąpała, Héctor Hernández-Lázaro, Agnieszka Sozańska

**Affiliations:** 1 Faculty of Health Sciences and Psychology, Collegium Medicum, University of Rzeszow, Rzeszow, 35-310 Poland; 2 Donum Corde Rehabilitation and Medical Care Center, Budy Głogowskie 835B Street, Głogów Małopolski, 36-060 Poland; 3 Faculty of Health Sciences, University of Valladolid, Soria, Spain; 4 Ólvega Primary Care Physiotherapy Unit, Soria Health Care Management, Castilla y León Regional Health Administration (SACYL), Soria, Spain

**Keywords:** Focal vibration therapy, Stroke, Rehabilitation, Motor function recovery

## Abstract

**Background:**

Early and intensive rehabilitation is particularly important for increasing neuroplasticity in patients after stroke. The aim of this study was to evaluate the effects of a 4-week rehabilitation program involving functional proprioceptive stimulation (FPS) on postural control and functional recovery in patients with stroke.

**Methods:**

This double-blinded, randomized controlled trial (RCT) was conducted at a tertiary care rehabilitation centre. Fifty patients with first stroke were recruited and randomly separated into a FPS Group (*n* = 25) or Sham Group (*n* = 25). Both groups underwent 3.5 h of rehabilitation, including physiotherapy, occupational therapy, verticalization and focal vibration on specific myotendinous junctions of the leg, for 5 days a week over 4 successive weeks. For the sham group, the focal vibrations had an amplitude of 0.1 mm with a fixed frequency of 40 Hz. For the FPS group, the stimulation consisted of focal vibrations of an amplitude of 2 mm with frequencies constantly changing between 40 and 85 Hz. Postural control evaluation was performed with the Berg Balance Scale (BBS), Functional Reach Test (FRT) and via the Alfa AC International East stabilometric platform; motor recovery was examined via the Barthel Index (BI) and ICF Rehabilitation Set. All assessments were performed at baseline and after intervention.

**Results:**

Following the intervention, the FPS Group demonstrated clinically significant improvements in postural control and functional status (BBS *p* = 0.041 and BI *p* = 0.037). A statistically significant improvement was also obtained in the Sham Group in the FTR test. Patients in the FPS Group experienced significantly greater improvement than those in the Sham Group on D420 (transferring oneself) and D640 (performing housework activities).

**Conclusions:**

Our study showed that conventional rehabilitation and rehabilitation combined with functional proprioceptive stimulation both improve balance and functional efficiency in people after.

**Trial registration:**

The study was registered at ClinicalTrials.gov (NCT05550987).

## Introduction

Stroke is a leading neurological disorder, affecting 15 million people globally each year a number expected to rise [1]. Poststroke disability is also increasing [2], with over 85% of survivors experiencing hemiplegia within 6–12 months, impairing mobility and independence [3]. Intensive rehabilitation during the acute phase of stroke can enhance spontaneous recovery [4] and actively stimulate neuroplasticity [5–7], thereby promoting more effective functional restoration. Optimal recovery, however, depends on a comprehensive assessment that integrates multiple dimensions of the patient’s condition including physical, psychological, environmental, social, and personal factors guided by the International Classification of Functioning, Disability and Health (ICF) framework.

The process of neuroplasticity involves molecular and cellular mechanisms that are responsible for the reconstruction of damaged neuronal networks [8]. This form of poststroke recovery involves axonal sprouting, tumorisation and strengthening of synapses, as well as compensatory mechanisms to restore lost functions [9]. The aim of early and intensive rehabilitation is to increase neuroplasticity in order to achieve better functional results [7]. Although several non-pharmacological methods are available to enhance the effects of therapy, such as electrotherapy, shockwave therapy, neuromodulation and stretching, evidence of their effectiveness is limited [10].

A promising and emerging method involves applying focal vibration near the muscle-tendon junction to elicit a kinaesthetic sensation of movement [11]. This type of stimulation is called functional proprioceptive stimulation (FPS). FPS activates muscle spindles, i.e., stretches receptors that signal changes in muscle length [12]. FPS at frequencies between 20 and 100 Hz induces a sensation of muscle stretching and causes the perception of movement [13]. It also increases the excitability of the sensorimotor cortex [14].

According to recent scientific reports, FPS has been shown to be safe, well tolerated, and easy to use. It can be an important complementary therapy for promoting motor recovery in patients who have recently had a stroke [15]. Wang et al. reported that vibration therapy effectively improves upper limb motor function in patients with subacute stroke. The effectiveness of sensory pathways is increased by the underlying mechanism of vibration, which reorganises and induces plastic changes in the motor and somatosensory cortex [16].

There is no consensus in the literature on the most effective rehabilitation protocol for improving the condition of poststroke patients. According to a review by Wang et al., further research is required to determine the optimal vibration parameters and dosage for FPS protocols in post-stroke patients [17]. Modern rehabilitation should incorporate both traditional methods and innovative technological solutions [18]. Therefore, it is crucial for specialists in clinical practice to identify the most effective rehabilitation method for helping poststroke patients regain lost function. Accordingly, the purpose of our study was to evaluate the effects of rehabilitation via functional proprioceptive stimulation, on top of conventional therapy, on postural control and functional performance in poststroke patients.

## Materials and methods

### Ethics

Ethical approval for this study was obtained from the University of Rzeszow Bioethics Committee (No. 2022/059). In accordance with the Declaration of Helsinki, all the subjects were informed about the purpose and procedure of the study and gave their informed consent to participate in the study.

### Design and setting

This was a double-blinded (participants and evaluators) randomized controlled trial. The research was carried out in a tertiary care rehabilitation centre between December 2022 and May 2024. The study was registered in the clinical trials register at ClinicalTrials.gov (NCT05550987) [19].

### Participants

Patients with first-ever ischaemic stroke were recruited from the rehabilitation centre. The inclusion criteria were as follows: (1) 18–65 years of age; (2) 2–6 months after onset; (3) ability to maintain an upright position (without assistance or with the help of a walker); and (4) the Abbreviated Mental Test Score > 6. The exclusion criteria were as follows: (1) severe comorbidities such as infections, cardiovascular or lung diseases; and (2) a spasticity level of the lower limbs of 4 on the Ashworth Scale. After taking into account the inclusion and exclusion criteria, 50 participants were included in the study (25 participants in each group).

### Qualification procedure for intervention

Patients who met the inclusion criteria were evaluated by a physician. Those eligible subjects were subsequently randomly assigned to one of two groups: the FPS group (VG) and the sham stimulation group (SG). Subjects in both groups underwent two examinations. The first examination took place on the day the patient was admitted to the rehabilitation centre, prior to the beginning of the rehabilitation program. The second examination was performed after the 4-week programme had been completed.

### Rehabilitation program

#### Conventional rehabilitation

The rehabilitation program was developed in accordance with the current recommendations of the European Stroke Organization and the American Heart Association [20, 21]. On the basis of these recommendations, the study group was provided with a comprehensive rehabilitation program of three and a half hours per day, which included one hour of individual therapy with a physiotherapist, one hour of verticalization, one hour of daily activity training, and 30 min of FPS or sham stimulation for five days (Monday through Friday) per week over a period of one month.

Individual therapy was conducted with a physiotherapist who had at least three years of experience in neurological physiotherapy. The therapy included work on the appropriate positioning of the patient in low and high positions. It also involved trunk stabilisation through functional tasks [22], work to normalise muscle tension in the trunk and limbs, and exercise to improve deep and superficial perception. Additionally, therapy also includes postural control exercises and body position change training [23]. These exercises consisted of shifting the centre of gravity forwards and shifting the body weight to the affected limb and gradually reducing the support plane, as well as performing exercises on an unstable surface [24].

The verticalisation was performed in a static parapodium. During the process, attention was given to ensuring the correct symmetry of the trunk and limbs. Simple exercises were performed to improve trunk stabilisation. The functional tasks performed during verticalisation were designed to deliver as much sensorimotor stimulation as possible to the patient’s damaged body segments [25].

Daily activities training focused on improving skills such as eating meals, dressing and using the toilet independently. In line with recommendations in poststroke rehabilitation, this therapy was conducted by an occupational therapist experienced in neurological rehabilitation. The therapist created safe conditions for training independence in daily activities, encouraged self-care, and introduced elements of family education [26].

During all individual therapies, the patients’ well-being, blood pressure, heart rate and saturation parameters were monitored.

#### Functional proprioceptive stimulation

**Fig. 1:**
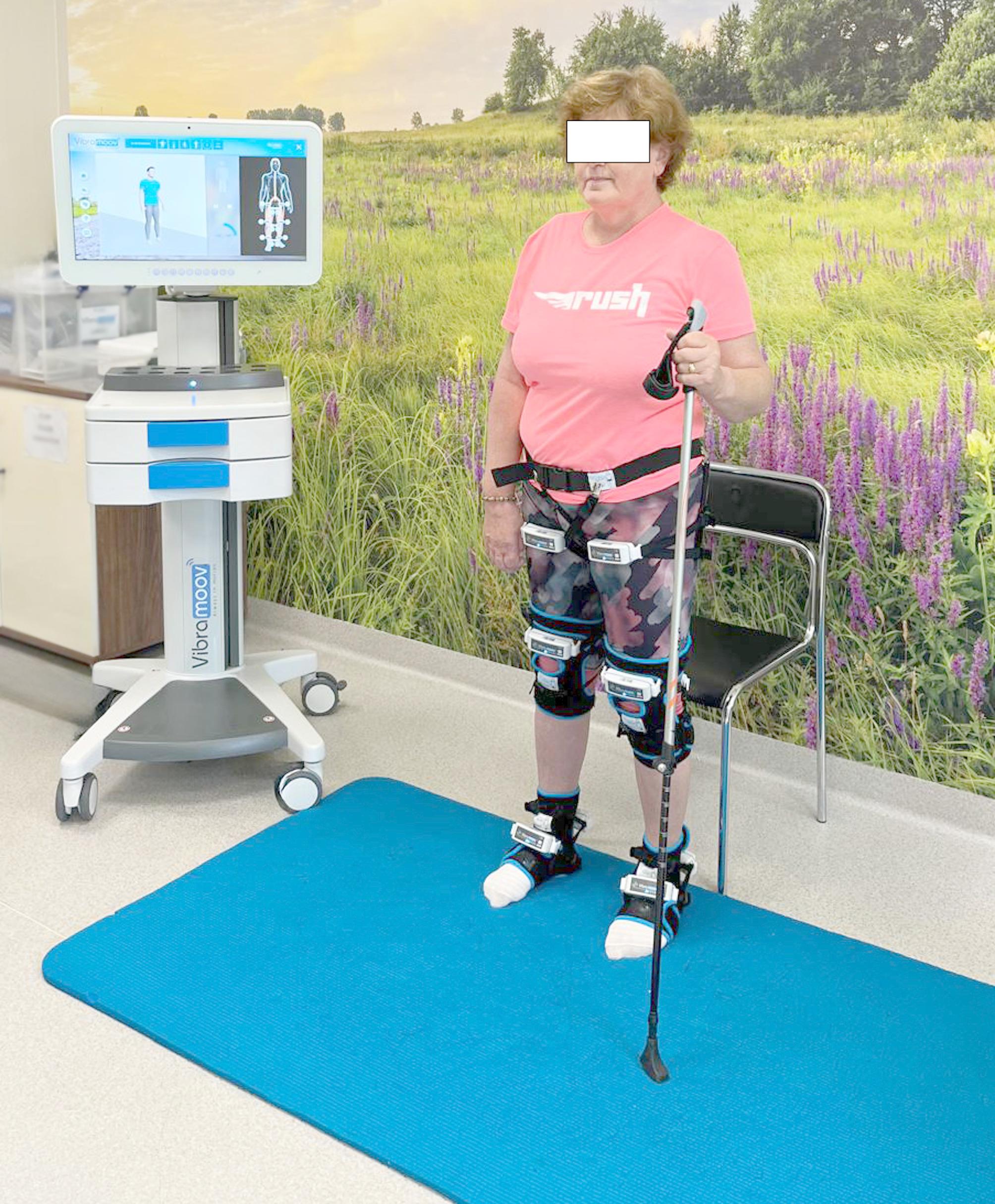
Functional proprioceptive stimulation session

Both groups received stimulation from the same device: Vibramoov PRO (Techno Concept, Manosque, France). Therapy based on FPS causes the patient to experience a kinaesthetic sensation of movement, which can consequently cause motor reactions corresponding to the movements experienced by the patient. In the Vibramoov device, focal vibrations are displayed using 12 wireless electromechanical vibrators attached to specific parts of the limbs with the help of dedicated orthoses. Stimulators were installed on standing patients at the muscle‒tendon junction located near the hip, knee and ankle joints. A specific program dedicated to improving postural control was systematically used. The patient was in a standing position throughout the therapy. In the first phase of the therapy, the patient was instructed to focus on the sensation of vibration in individual muscle groups without the possibility of visually tracking the movement on the monitor. In the next phase, the patient’s task was to engage in the movement stimulated by the FPS (forwards/backwards disequilibrium; rotation of the trunk to the right and left). In the last phase, the patient was able to look at a visual avatar mimicking the supposed kinaesthetic sensation felt. The patient’s task was to reproduce in detail the movements programmed in the device, stimulated by FPS, and simultaneously visually control the video avatar on the screen of the control unit (Figure 1) [11].

In the VG group, FPS with an amplitude of 1–2 mm and frequencies of 40–85 Hz were used, whereas in the SG group, FPS with an amplitude of 0.1 mm and a frequency of 40 Hz was used. The parameters used in the SG group are below the minimum amplitude required to induce kinesthetic illusion [27]. The parameters adopted were related to the specificity of the device used in the intervention. Both stimulation procedures were performed in the same way, which lasted 30 min according to the literature review by Fattorini et al. [28].

### Data collection

An interview using a questionnaire was used to collect information on sociodemographic variables: age, sex and place of residence. Data on the type of stroke (haemorrhagic/ischaemic) and the time since the incident were obtained from the patients’ medical records.

## Measurements

### Primary outcome

#### Postural control assessment

##### The berg balance scale (BBS)

The BBS was used to assess functional balance. The assessment was conducted in 14 tests, which refer to simple daily activities performed in a sitting and standing position, including balance while sitting down, standing up and sitting down, reaching, picking up objects from the floor or climbing a step. The level of balance in each of the tests was assessed on a 4-point scale, where 4 means full independence in performing the test and 0 means complete inability to perform it. The maximum possible score on the scale was 56 points, representing the total sum of the individual test results [29, 30]. As demonstrated by Myiata et al., among others, the BBS is a reliable tool for assessing the balance and postural stability of patients after stroke (Cronbach’s alpha– 0.97). It supports the setting of therapy goals and the planning of clinical interventions [31].

### Secondary outcomes

#### Trunk Impairment Scale (TIS)

The TIS was used to assess trunk function, and its reliability and validity for use with stroke patients have been confirmed in many clinical studies (Cronbach’s alpha greater than 0.80) [32, 33].The scale evaluates the patient’s trunk function in 17 tests related to static balance in a sitting position, dynamic balance in a sitting position, and coordination. The maximum score that can be achieved on the scale is 23 points [34].

#### Functional Reach Test (FRT)

The FRT evaluates trunk stability and dynamic balance when standing, with the trunk tilting in the sagittal plane. Numerous studies have demonstrated the high reliability of this measure (Interclass Correlattion Coefficient  = 0.83–0.87) and its significant correlation with body’s centre of gravity (COP) tilt [35, 36]. The subject was positioned sideways to the wall, ensuring they did not lean against it. They were asked to extend their arms straight at the elbow (or, if it was impossible to raise both arms, only the uninvolved arm) to a height of 90 degrees. In this position, the examiner marked the starting position on the tape measure located on the wall. Then, the subject was asked to flex their hips without lifting their feet from the ground and move their torso forwards. In the position of maximum inclination, the examiner noted the position of the patient’s third metacarpal bones. The difference in centimetres between the first and second values on the tape measure was the result of the FRT test. The test was performed in 3 trials, with the first trial serving as a demonstration and providing instructions on how to perform the test. The recorded result was the average of the last two trials [37, 38].

#### Postural stability

Static balance and postural stability was assessed using the Alfa AC International East stabilometric platform (Technomex Sp. z.o.o.). Subjects were asked to stand on the platform with their upper limbs hanging loosely by their sides and their feet hip-width apart. The best foot position was recorded in the study protocol to enable its reproduction during the second study. The test was conducted twice, once with the eyes open and once the eyes closed. Computer analysis yielded the following parameters for open and closed eyes: the length of the path travelled by the body’s centre of gravity (PL); the area defined by the body’s centre of gravity (S); the deviation of the body’s centre of gravity in the X-axis (XD), the deviation of the body’s centre of gravity in the Y-axis (YD), the average velocity of the body’s centre of gravity in the X-axis (ASX) and the average velocity of the body’s centre of gravity in the Y-axis (ASY) [39, 40].

#### Functional status assessment

##### Barthel Index (BI)

The BI evaluates a patient’s ability to perform basic daily activities. The scale includes 10 activities, such as dressing, personal hygiene, bathing, moving around flat surfaces and stairs, eating, using the toilet, and controlling the excretion of urine and stool. Depending on the degree of independence in each of these activities, the patient can receive 5, 10 or 15 points. A total score of 100 points indicates the patient’s full independence in basic daily activities [41]. The reliability of BI in patients after stroke ranges from moderate to very good [42].

##### ICF Rehabilitation Set

Core sets are tools that contain lists of basic categories relevant to specific conditions. These tools were developed on the basis of the International Classification of Functioning, Disability and Health (ICF), and their form allows for use in everyday clinical practice [43, 44]. The study used the Polish version of the ICF rehabilitation set validated by Wiśniowska-Szurlej et al. The set consists of 29 categories, including 8 categories for body functions and structures (B130, B134, B152, B280, B455, B620, B710, and B730), 16 categories for activity and participation (D230, D240, D410, D415, D420, D445, D450, D465, D510, D530, D540, D550, D570, D640, D710, D850, and D920) and 5 categories for environmental factors (E110, E115, E155, E310, and E450). The individual categories were assessed according to the same 0–4 scale proposed by the WHO [45]. Scientific studies to date have confirmed the benefits of using a uniform, international system for defining various clinical conditions of patients [46, 47].

#### Sample size

The sample size was determined based on the results of the pilot study. Given that balance disorders are a common dysfunction in stroke patients, we calculated the sample size based on the results of the BBS (our primary outcome). We assumed that the BBS score would increase by an average of 15.0 points in the control group and by 18.9 points in the treatment group. The common standard deviation for both groups, estimated in the pilot study, was approximately σ = 4.0 points. We set a two-sided significance level of α = 0.05 and a power of the test of 1 − β = 0.90 (corresponding to β = 0.1). The difference in improvement between the groups (δ) that we intended to detect was therefore assumed to be 18.9 − 15.0 = 3.9 points. Given these assumptions, the critical values from the normal distribution were z_α/2_ = 1.96 and z_β_ = 1.282. After substituting these values into the standard sample size formula for comparing two means, $\:n1=n2=2{\left[\frac{\left({z}_{\alpha\:/2}+{z}_{\beta\:}\right)\sigma\:}{\delta\:}\right]}^{2}$, the required minimum number of participants n_1_ = n_2_≈22 was obtained in each of the study groups (the exact calculation for σ = 3.96 and δ = 3.9 gives *n*≈21.67). Taking into account a possible 10% withdrawal rate, it was decided to include 25 participants in each group, giving a total required number of 50 participants in the study.

#### Randomization

Randomisation was implemented using the stratified method in the R statistical package 4.4.1 (The. R Foundation for Statistical Computing, Vienna, Austria). One-in-one blocks were randomised, which made it possible to obtain an even distribution of patients in the study groups. The randomisation order was determined using a computerised random numbers schedule. Randomisation was implemented by an independent biostatistician, who hid the block size from the executive module and used randomly mixed block sizes.

#### Blinding

A double-blind design was used for this study. The study participants were unaware of whether they were in the FPS group VG group or the SG group. Futhermore, those taking the final measurements were unaware of group allocation and did not participate in the intervention’s implementation.

### Statistical analysis

Quantitative data are expressed as means, standard deviations, medians, quartiles and ranges. Qualitative variables are expressed as absolute and relative frequencies (N and %). The chi-square test (with Yates correction for 2 × 2 tables) or Fisher’s exact test (in the case of low expected values) was used to compare qualitative variables between groups. The Mann‒Whitney test was used to compare quantitative variables between two groups. A paired Wilcoxon test was used to compare two repeated measures of quantitative variables. The Bhapkar test was used to compare repeated measures of qualitative variables. The significance level was set to 0.05. All the analyses were conducted using R software, version 4.4.1.

## Results

**Fig. 2:**
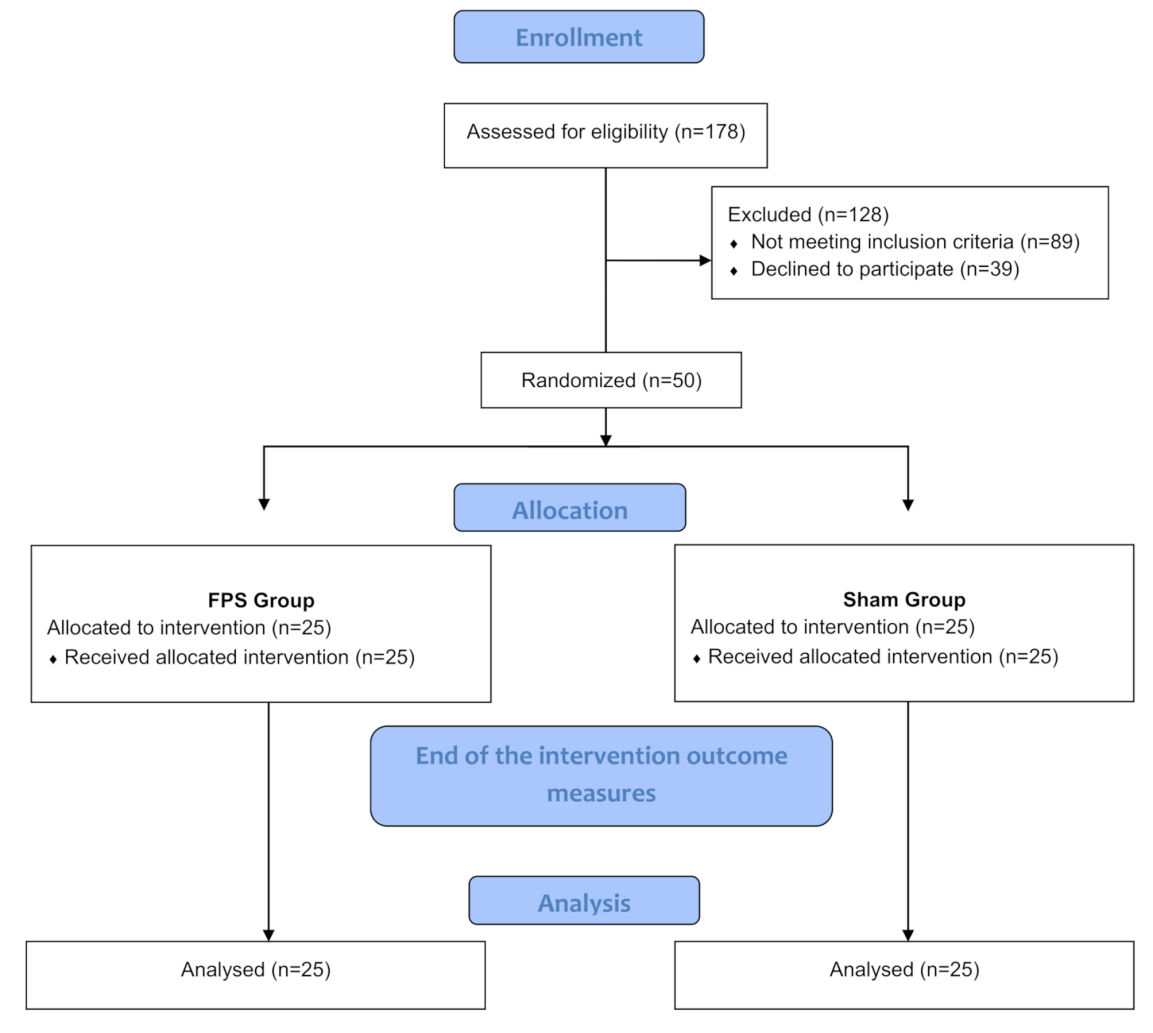
Flow diagram for study participants

The study included 178 patients after ischaemic stroke. After a preliminary examination was conducted and the inclusion and exclusion criteria were applied, 50 patients (35 men and 15 women) were included in the analysis. The mean age of the study participants was 57.46 years (± 13.09). On average, 4.64 (± 1.03) years had elapsed since the stroke incident. Patients who qualified for the study were assigned to two groups: the VG group (*n* = 25) and the SG group (*n* = 25). Figure 2 shows the CONSORT flow diagram of the study participants.

During the study, none of the patients experienced any adverse events. The final analysis included patients with an exercise attendance rate of was over 95%. There were no differences baseline parameters among the study groups in terms of sociodemographic characteristics, cognitive status, functional status or body balance. The baseline results of the studied variables before the intervention are presented in Table 1.

**Table 1:** Baseline characteristics of the 2 groups before the intervention

Parameter		FPS Group	Sham Group	Total Group	*p*
**Sociodemographic**		Mean (95% CI), Number (%)			
**Gender**	Female	8 (32.00)	7 (28.00)	15 (30.00)	*p* = 1.000
	Male	17 (68.00)	18 (72.00)	35 (70.00)	
	**BMI [kg/m²]**	26.90 (4.15)	27.63 (3.24)	27.27 (3.7)	*p* = 0.522
	**Age [years]**	59.00 (11.38)	55.92 (14.67)	57.46 (13.09)	*p* = 0.634
	**Months** **since stroke**	4.60 (1.00)	4.68 (1.07)	4.64 (1.03)	*p* = 0.786
	**AMTS**	7.96 (7.20–8.72)	7.80 (7.06–8.54)	7.88 (7.35–8.41)	*p* = 0.722
**Primary Outcome**		Mean (95% CI)			
***Postural control assessment***					
	BBS	29.00 (24.71–33.29)	28.08 (23.06–33.10)	28.54 (25.27–31.81)	*p* = 0.801
**Secondary Outcome**		Mean (95% CI)			
	TIS	14.84 (13.17–16.51)	14 (12.47–15.53)	14.42 (13.3–15.54)	*p* = 0.459
	FRT	13.24 (9.59–16.89)	14.48 (11.23–17.73)	13.86 (11.43–16.29)	*p* = 0.331
**Postural Balance Assessment (eyes open)**	PL	54.01 (40.17–67.85)	54.88 (41.89–67.87)	54.45 (45.06–63.84)	*p* = 0.669
	S	7.70 (4.8–10.6)	9.05 (4.58–13.52)	8.37 (5.72–11.02)	*p* = 0.577
	YD	0.59 (-0.43–1.61)	-0.18 (-1.12–0.76)	0.21 (-0.48–0.90)	*p* = 0.415
	XD	0.62 (-0.26–1.5)	-0.67 (-1.41–0.07)	-0.02 (-0.63–0.59)	*p* = 0.071
	ASY	1.12 (0.81–1.43)	0.80 (0.51–1.09)	0.96 (0.74–1.18)	*p* = 0.449
	ASX	0.90 (0.41–1.39)	0.85 (0.63–1.07)	0.88 (0.63–1.13)	*p* = 0.628
**Postural Balance Assessment (eyes closed)**	PL	82.53 (61.85–103.21)	77.32 (39.22–115.42)	79.92 (58.46–101.38)	*p* = 0.244
	S	13.18 (8.10–18.26)	13.82 (8.04–19.6)	13.50 (9.7–17.3)	*p* = 0.720
	YD	0.92 (-0.08–1.92)	-0.10 (-1.06–0.86)	0.41 (-0.30–1.12)	*p* = 0.211
	XD	0.79 (-0.17–1.75)	-0.55 (-1.41–0.31)	0.12 (-0.55–0.79)	*p* = 0.121
	ASY	1.86 (1.39–2.33)	1.39 (0.82–1.96)	1.62 (1.25–1.99)	*p* = 0.057
	ASX	1.32 (0.65–1.99)	1.47 (0.39–2.55)	1.39 (0.76–2.02)	*p* = 0.135
***Functional status assessment***					
	BI	68.60 (61.03–76.17)	65.00 (56.91–73.09)	66.80 (61.29–72.31)	*p* = 0.585
**ICF Rehabilitation Set**	B130	1.56 (1.23–1.89)	1.84 (1.45–2.23)	1.70 (1.45–1.95)	*p* = 0.292
	B134	1.04 (0.69–1.39)	1.56 (1.19–1.93)	1.30 (1.05–1.55)	*p* = 0.053
	B152	1.68 (1.31–2.05)	2.00 (1.57–2.43)	1.84 (1.57–2.11)	*p* = 0.270
	B280	1.36 (0.89–1.83)	1.72 (1.25–2.19)	1.54 (1.21–1.87)	*p* = 0.293
	B455	1.56 (1.29–1.83)	1.92 (1.55–2.29)	1.74 (1.50–1.98)	*p* = 0.137
	B620	0.96 (0.59–1.33)	1.2 (0.85–1.55)	1.08 (0.83–1.33)	*p* = 0.363
	B710	1.88 (1.59–2.17)	1.92 (1.59–2.25)	1.90 (1.68–2.12)	*p* = 0.846
	B730	2.40 (2.16–2.64)	2.28 (1.89–2.67)	2.34 (2.12–2.56)	*p* = 0.600
	D230	1.84 (1.45–2.23)	2.04 (1.65–2.43)	1.94 (1.67–2.21)	*p* = 0.484
	D240	1.80 (1.47–2.13)	1.84 (1.49–2.19)	1.82 (1.58–2.06)	*p* = 0.873
	D415	1.12 (0.73–1.51)	1.32 (0.95–1.69)	1.22 (0.95–1.49)	*p* = 0.474
	D410	1.36 (1.03–1.69)	1.32 (1.01–1.63)	1.34 (1.10–1.58)	*p* = 0.866
	D420	1.32 (0.91–1.73)	1.36 (1.01–1.71)	1.34 (1.07–1.61)	*p* = 0.887
	D450	2.28 (1.89–2.67)	2.44 (2.01–2.87)	2.36 (2.07–2.65)	*p* = 0.594
	D465	2.04 (1.47–2.61)	2.16 (1.65–2.67)	2.10 (1.73–2.47)	*p* = 0.758
	D510	1.68 (1.27–2.09)	2.00 (1.49–2.51)	1.84 (1.51–2.17)	*p* = 0.337
	D530	1.08 (0.73–1.43)	1.64 (1.17–2.11)	1.36 (1.07–1.65)	*p* = 0.067
	D540	1.68 (1.35–2.01)	1.84 (1.43–2.25)	1.76 (1.51–2.01)	*p* = 0.552
	D550	0.68 (0.39–0.97)	1.4 (0.93–1.87)	1.04 (0.75–1.33)	*p* = 0.014
	D570	2.04 (1.55–2.53)	2.44 (1.97–2.91)	2.24 (1.91–2.57)	*p* = 0.251
	D640	2.72 (2.29–3.15)	2.72 (2.27–3.17)	2.72 (2.41–3.03)	*p* = 1,000
	D710	1.40 (0.93–1.87)	1.64 (1.13–2.15)	1.52 (1.17–1.87)	*p* = 0.503
	D850	3.48 (3.17–3.79)	3.16 (2.67–3.65)	3.32 (3.03–3.61)	*p* = 0.290
	D920	2.28 (1.77–2.79)	2.36 (1.93–2.79)	2.32 (1.99–2.65)	*p* = 0.817
	E110	-1.72 (-2.58 - -0.86)	-1.2 (-2.02 - -0.38)	-1.46 (-2.05 - -0.87)	*p* = 0.398
	E115	-1.56 (-2.54 - -0.58)	-1.64 (-2.60 - -0.68)	-1.60 (-2.29 - -0.91)	*p* = 0.910
	E155	0.92 (0.14–1.70)	0.16 (-0.86–1.18)	0.54 (-0.11–1.19)	*p* = 0.253
	E310	-1.92 (-2.78 - -1.06)	-1.28 (-2.16 - -0.40)	-1.60 (-2.21 - -0.99)	*p* = 0.309
	E450	-0.56 (-1.19–0.07)	-0.44 (-0.97–0.09)	-0.50 (-0.91 - -0.09)	*p* = 0.777

BMI– body mass index; AMTS– Abbreviated Mental Test Score; BBS– Berg Balance Scale, TIS– Trunk Impairment Scale; FRT– Functional Reach Test; PL - center of gravity; S– area; XD - deviation in the X axis; YD - deviation in the X axis; ASX - average velocity in the X axis; ASY - average velocity in the Y axis; BI–Barthel Index

After 4 weeks of exercise, both the VG and SG groups showed a statistically significant improvement in functional balance, as assessed via the BBS test (VG *p* < 0.001 vs. SG *p* = 0.001). A significant improvement was also demonstrated in the VG group in terms of functional ability (BI). In the SG group, only the ability expressed by the BI test improved significantly (*p* = 0.003). In the VG group, the COP path length decreased significantly, with both eyes open and closed. The VG group also showed a statistically significant improvement in body function, activity and participation, as assessed via the ICF Rehabilitation Set entities (Table 2).

**Table 2:** Data before and after the intervention for the FPS group and the Sham group

	FPS Group			Sham Group			
**Parameter**	**Before rehabilitation**	**After rehabilitation**		**Before rehabilitation**		**After rehabilitation**	
	**Mean (95CI)**		**p**	**Mean (95CI)**			**p**
**Primary Outcome**							
***Postural control assessment***							
BBS	29.00 (24.71–33.29)	38.40 (34.75–42.05)	*p* < 0.001 *	28.08 (23.06–33.1)		34.00 (30.14–37.86)	*p* = 0.001 *
**Secondary Outcome**							
TIS	14.84 (13.17–16.51)	16.32 (14.42–18.22)	*p* = 0.068	14.00 (12.47–15.53)		13.28 (11.44–15.12)	*p* = 0.866
FRT	13.24 (9.59–16.89)	18.52 (15.56–21.48)	*p* < 0.001 *	14.48 (11.23–17.73)		16.72 (13.72–19.72)	*p* = 0.003 *
**Postural Balance Assessment (eyes open)**							
PL	54.01 (40.17–67.85)	39.46 (31.35–47.57)	*p* = 0.033 *	54.88 (41.89–67.87)		53.66 (41.63–65.69)	*p* = 0.537
S	7.70 (4.80–10.60)	11.57 (4.91–18.23)	*p* = 0.843	9.05 (4.58–13.52)		6.58 (2.72–10.44)	*p* = 0.135
YD	0.59 (-0.43–1.61)	0.73 (-0.35–1.81)	*p* = 0.964	-0.18 (-1.12–0.76)		-0.10 (-0.83–0.63)	*p* = 0.846
XD	0.62 (-0.26–1.5)	0.34 (-0.52–1.20)	*p* = 0.465	-0.67 (-1.41–0.07)		-0.14 (-0.71–0.43)	*p* = 0.118
ASY	1.12 (0.81–1.43)	1.09 (0.78–1.40)	*p* = 0.570	0.80 (0.51–1.09)		0.74 (0.45–1.03)	*p* = 0.330
ASX	0.90 (0.41–1.39)	0.96 (0.71–1.21)	*p* = 0.237	0.85 (0.63–1.07)		0.78 (0.56–1.00)	*p* = 0.305
**Postural Balance Assessment (eyes closed)**							
PL	82.53 (61.85–103.21)	56.64 (44.19–69.09)	*p* = 0.017 *	77.32 (39.22–115.42)		65.22 (47.17–83.27)	*p* = 0.603
S	13.18 (8.10–18.26)	9.97 (5.87–14.07)	*p* = 0.080	13.82 (8.04–19.6)		6.53 (3.35–9.71)	*p* = 0.015 *
YD	0.92 (-0.08–1.92)	1.24 (0.14–2.34)	*p* = 0.922	-0.1 (-1.06–0.86)		0.11 (-0.67–0.89)	*p* = 0.821
XD	0.79 (-0.17–1.75)	0.41 (-0.43–1.25)	*p* = 0.399	-0.55 (-1.41–0.31)		-0.32 (-0.97–0.33)	*p* = 0.677
ASY	1.86 (1.39–2.33)	1.55 (1.18–1.92)	*p* = 0.346	1.39 (0.82–1.96)		1.22 (0.91–1.53)	*p* = 0.945
ASX	1.32 (0.65–1.99)	1.26 (0.97–1.55)	*p* = 0.475	1.47 (0.39–2.55)		1.02 (0.77–1.27)	*p* = 0.972
***Functional status assessment***							
BI	68.60 (61.03–76.17)	83.80 (77.78–89.82)	*p* < 0.001 *	65.00 (56.91–73.09)		73.40 (65.87–80.93)	*p* = 0.003 *
**ICF Rehabilitation Set**							
B130	1.56 (1.23–1.89)	1.16 (0.85–1.47)	*p* = 0.005 *	1.84 (1.45–2.23)	1.56 (1.21–1.91)		*p* = 0.011 *
B134	1.04 (0.69–1.39)	0.76 (0.45–1.07)	*p* = 0.011 *	1.56 (1.19–1.93)	1.08 (0.73–1.43)		*p* = 0.002 *
B152	1.68 (1.31–2.05)	1.24 (0.91–1.57)	*p* = 0.003 *	2.00(1.57–2.43)	1.60 (1.19–2.01)		*p* = 0.014 *
B280	1.36 (0.89–1.83)	0.88 (0.57–1.19)	*p* = 0.002 *	1.72 (1.25–2.19)	1.08 (0.71–1.45)		*p* < 0.001 *
B455	1.56 (1.29–1.83)	1.24 (0.97–1.51)	*p* = 0.006 *	1.92 (1.55–2.29)	1.56 (1.31–1.81)		*p* = 0.008 *
B620	0.96 (0.59–1.33)	0.52 (0.28–0.76)	*p* = 0.001 *	1.2 (0.85–1.55)	0.92 (0.57–1.27)		*p* = 0.066
B710	1.88 (1.59–2.17)	1.42 (1.18–1.66)	*p* = 0.003 *	1.92 (1.59–2.25)	1.64 (1.37–1.91)		*p* = 0.039 *
B730	2.40 (2.16–2.64)	1.92 (1.67–2.17)	*p* = 0.001 *	2.28 (1.89–2.67)	1.84 (1.51–2.17)		*p* = 0.001 *
D230	1.84 (1.45–2.23)	1.36 (1.01–1.71)	*p* = 0.002 *	2.04 (1.65–2.43)	1.52 (1.15–1.89)		*p* < 0.001 *
D240	1.8 0(1.47–2.13)	1.32 (0.97–1.67)	*p* = 0.002 *	1.84 (1.49–2.19)	1.48 (1.13–1.83)		*p* = 0.023 *
D415	1.12 (0.73–1.51)	0.48 (0.23–0.73)	*p* < 0.001 *	1.32 (0.95–1.69)	1.00 (0.69–1.31)		*p* = 0.115
D410	1.36 (1.03–1.69)	0.76 (0.49–1.03)	*p* = 0.001 *	1.32 (1.01–1.63)	1.04 (0.71–1.37)		*p* = 0.023 *
D420	1.32 (0.91–1.73)	0.76 (0.43–1.09)	*p* < 0.001 *	1.36 (1.01–1.71)	1.32 (0.91–1.73)		*p* = 0.842
D450	2.28 (1.89–2.67)	1.84 (1.51–2.17)	*p* = 0.009 *	2.44 (2.01–2.87)	1.84 (1.39–2.29)		*p* = 0.001 *
D465	2.04 (1.47–2.61)	1.52 (1.13–1.91)	*p* = 0.004 *	2.16 (1.65–2.67)	1.52 (0.99–2.05)		*p* = 0.001 *
D510	1.68 (1.27–2.09)	1.40 (1.05–1.75)	*p* = 0.039 *	2.00 (1.49–2.51)	1.48 (1.07–1.89)		*p* = 0.002 *
D530	1.08 (0.73–1.43)	0.76 (0.45–1.07)	*p* = 0.006 *	1.64 (1.17–2.11)	1.12 (0.75–1.49)		*p* = 0.001 *
D540	1.68 (1.35–2.01)	1.32 (1.07–1.57)	*p* = 0.003 *	1.84 (1.43–2.25)	1.52 (1.17–1.87)		*p* = 0.006 *
D550	0.68 (0.39–0.97)	0.44 (0.20–0.68)	*p* = 0.020 *	1.40 (0.93–1.87)	1.00 (0.73–1.27)		*p* = 0.056
D570	2.04 (1.55–2.53)	1.64 (1.21–2.07)	*p* = 0.002 *	2.44 (1.97–2.91)	1.76 (1.29–2.23)		*p* = 0.001 *
D640	2.72 (2.29–3.15)	2.08 (1.69–2.47)	*p* = 0.001 *	2.72 (2.27–3.17)	2.44 (1.91–2.97)		*p* = 0.026 *
D710	1.40 (0.93–1.87)	1.00 (0.61–1.39)	*p* = 0.005 *	1.64 (1.13–2.15)	1.16 (0.69–1.63)		*p* = 0.004 *
D850	3.48 (3.17–3.79)	3.12 (2.67–3.57)	*p* = 0.003 *	3.16 (2.67–3.65)	2.80 (2.25–3.35)		*p* = 0.098
D920	2.28 (1.77–2.79)	1.76 (1.23–2.29)	*p* = 0.002 *	2.36 (1.93–2.79)	2.00 (1.49–2.51)		*p* = 0.015 *
E110	-1.72 (-2.58 - -0.86)	-1.48 (-2.34 - -0.62)	*p* = 0.786	-1.2 (-2.02 - -0.38)	-1.16 (-2.02 - -0.3)		*p* = 1
E115	-1.56 (-2.54 - -0.58)	-1.20 (-2.18 - -0.22)	*p* = 0.584	-1.64 (-2.6 - -0.68)	-1.60 (-2.54 - -0.66)		*p* = 1
E155	0.92 (0.14–1.70)	1.12 (0.59–1.65)	*p* = 0.582	0.16 (-0.86–1.18)	0.80 (-0.02–1.62)		*p* = 0.203
E310	-1.92 (-2.78 - -1.06)	-1.24 (-2.08 - -0.40)	*p* = 0.106	-1.28 (-2.16 - -0.4)	-1.20 (-2.06 - -0.34)		*p* = 0.751
E450	-0.56 (-1.19–0.07)	-0.64 (-1.25 - -0.03)	*p* = 0.424	-0.46 (-1.01–0.09)	-0.67 (-1.22 - -0.12)		*p* = 0.212

BBS– Berg Balance Scale, TIS– Trunk Impairment Scale; BI–I Barthel Index; FRT– Functional Reach Test; PL - center of gravity; S– area; XD - deviation in the X axis; YD - deviation in the X axis; ASX - average velocity in the X axis; ASY - average velocity in the Y axis; *- *p* < 0.05

**Fig. 3:**
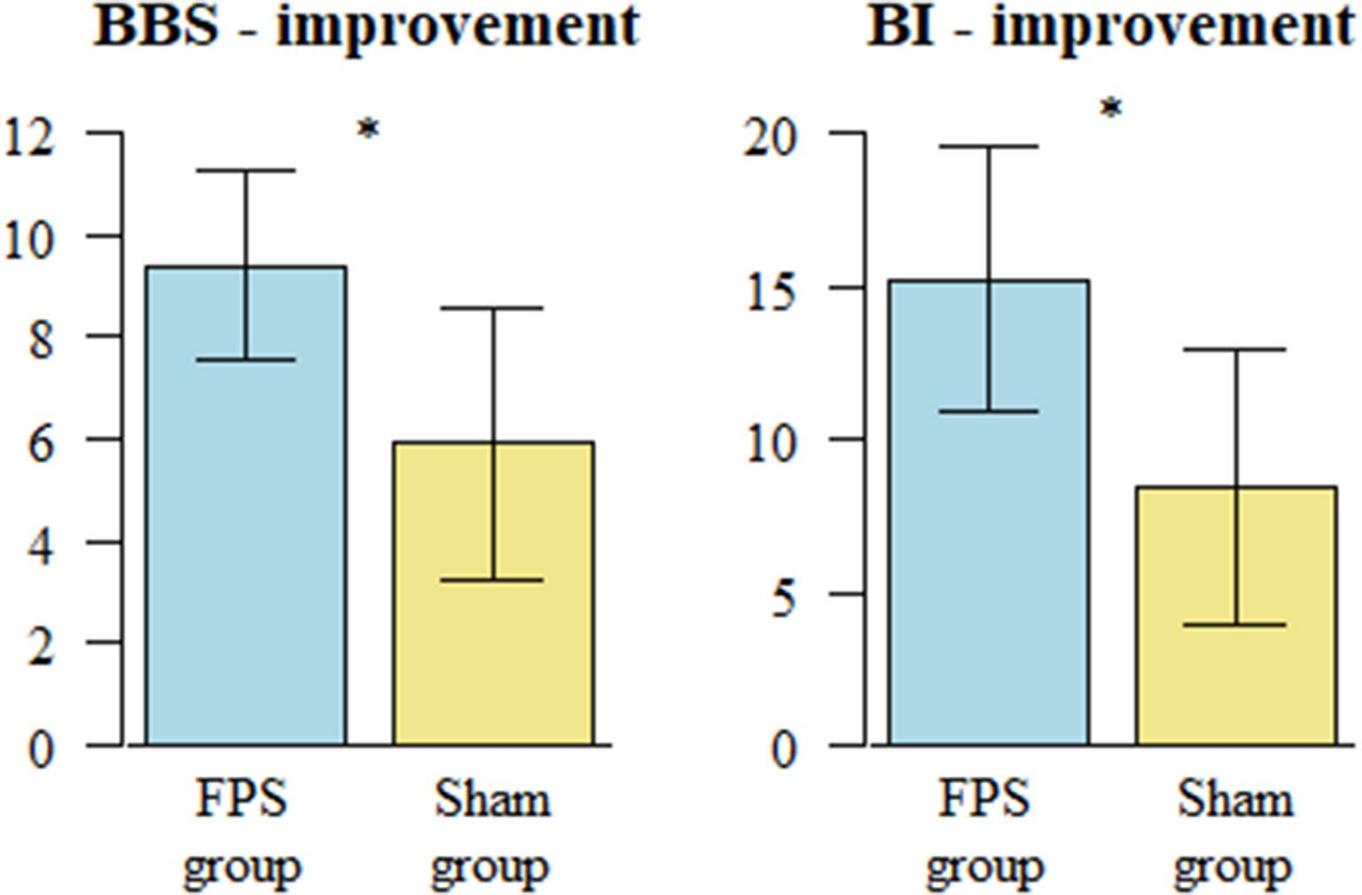
Comparison of functional outcome improvement between FPS group and the Sham group

Following the intervention, there was a significantly greater improvement in functional balance in the VG group (*p* = 0.041). The VG group achieved a mean increase of 9.4 points on the BBS scale, compared to 5.92 points in the SG group. Statistically significant improvements were also obtained in the SG group in the following tests: BI (15.12 in the VG group vs. 8.40 in the SG group) and FRT (5.28 in the VG group vs. 2.23 in the SG group). There was no difference in the improvement in COP path length between the studied groups. Patients in the VG group improved significantly more than those in the SG group on D420 (transferring oneself) and D640 (performing housework activities) (see Table 3; Fig. 3).

**Table 3:** Evaluation of the size of effects of the intervention for the FPS group and the Sham group

	FPS Group	Sham Group	
**Parameter**	**Mean (95CI)**		**p**
**Primary Outcome**			
***Postural control assessment***			
BBS	9.40 (7.52–11.28)	5.92 (3.25–8.59)	*p* = 0.041 *
**Secondary Outcome**			
TIS	1.48 (0.09–2.87)	-0.72 (-2.92–1.48)	*p* = 0.104
FRT	5.28 (3.97–6.59)	2.23 (0.78–3.68)	*p* = 0.004 *
**Postural Balance Assessment (eyes open)**			
PL	14.56 (2.06–27.06)	1.22 (-11.28–13.72)	*p* = 0.146
S	-3.88 (-9.41–1.65)	2.46 (-1.52–6.44)	*p* = 0.074
YD	-0.14 (-1.36–1.08)	-0.08 (-0.77–0.61)	*p* = 0.931
XD	0.28 (-0.52–1.08)	-0.53 (-1.2–0.14)	*p* = 0.139
ASY	0.03 (-0.21–0.27)	0.06 (-0.10–0.22)	*p* = 0.881
ASX	-0.06 (-0.47–0.35)	0.06 (-0.10–0.22)	*p* = 0.583
**Postural Balance Assessment (eyes closed)**			
PL	25.89 (8.35–43.43)	12.1 (-13.81–38.01)	*p* = 0.392
S	3.21 (-2.65–9.07)	7.28 (0.36–14.2)	*p* = 0.384
YD	-0.32 (-1.42–0.78)	-0.21 (-1.03–0.61)	*p* = 0.882
XD	0.38 (-0.40–1.16)	-0.23 (-0.96–0.50)	*p* = 0.268
ASY	0.31 (-0.10–0.72)	0.17 (-0.34–0.68)	*p* = 0.672
ASX	0.06 (-0.55–0.67)	0.45 (-0.59–1.49)	*p* = 0.531
***Functional status assessment***			
BI	15.20 (10.87–19.53)	8.40 (3.91–12.89)	*p* = 0.037 *
**ICF Rehabilitation Set**			
B130	0.40 (0.16–0.64)	0.28 (0.10–0.46)	*p* = 0.420
B134	0.28 (0.1–0.46)	0.48 (0.24–0.72)	*p* = 0.185
B152	0.44 (0.2–0.68)	0.40 (0.13–0.67)	*p* = 0.828
B280	0.48 (0.24–0.72)	0.64 (0.44–0.84)	*p* = 0.300
B455	0.32 (0.12–0.52)	0.36 (0.14–0.58)	*p* = 0.789
B620	0.44 (0.24–0.64)	0.28 (-0.07–0.63)	*p* = 0.439
B710	0.46 (0.22–0.7)	0.28 (0.04–0.52)	*p* = 0.305
B730	0.48 (0.28–0.68)	0.44 (0.24–0.64)	*p* = 0.782
D230	0.48 (0.24–0.72)	0.52 (0.32–0.72)	*p* = 0.798
D240	0.48 (0.24–0.72)	0.36 (0.09–0.63)	*p* = 0.514
D415	0.64 (0.39–0.89)	0.32 (-0.05–0.69)	*p* = 0.167
D410	0.60 (0.35–0.85)	0.28 (0.06–0.5)	*p* = 0.064
D420	0.56 (0.36–0.76)	0.04 (-0.35–0.43)	*p* = 0.022 *
D445	0.56 (0.31–0.81)	0.24 (-0.11–0.59)	*p* = 0.150
D450	0.44 (0.17–0.71)	0.60 (0.35–0.85)	*p* = 0.409
D465	0.52 (0.25–0.79)	0.64 (0.37–0.91)	*p* = 0.551
D510	0.28 (0.04–0.52)	0.52 (0.27–0.77)	*p* = 0.187
D530	0.32 (0.12–0.52)	0.52 (0.28–0.76)	*p* = 0.192
D540	0.36 (0.16–0.56)	0.32 (0.12–0.52)	*p* = 0.771
D550	0.24 (0.06–0.42)	0.4 (0.05–0.75)	*p* = 0.433
D570	0.40 (0.20–0.60)	0.68 (0.41–0.95)	*p* = 0.107
D640	0.64 (0.37–0.91)	0.28 (0.06–0.5)	*p* = 0.048 *
D710	0.4 (0.16–0.64)	0.48 (0.23–0.73)	*p* = 0.648
D850	0.36 (0.16–0.56)	0.36 (-0.01–0.73)	*p* = 1.000
D920	0.52 (0.27–0.77)	0.36 (0.11–0.61)	*p* = 0.385
E110	-0.24 (-0.91–0.43)	-0.04 (-0.33–0.25)	*p* = 0.594
E115	-0.36 (-1.12–0.4)	-0.04 (-0.41–0.33)	*p* = 0.464
E155	-0.2 (-0.69–0.29)	-0.64 (-1.5–0.22)	*p* = 0.392
E310	-0.68 (-1.39–0.03)	-0.08 (-0.47–0.31)	*p* = 0.151
E450	0.08 (-0.08–0.24)	0.21 (-0.1–0.52)	*p* = 0.469

## Discussion

Despite the availability of therapies and innovative preventive strategies, stroke remains one of the leading causes of disability worldwide [48]. Patients who have had a stroke experience complex combination of motor, sensory, emotional and cognitive deficits that can affect static and dynamic balance and functional ability [49]. Given that repetitive sensory stimuli are among the most effective modulators of somatosensory and corticomotor structures, FPS is becoming increasingly popular as.

a well-tolerated, safe and non-invasive technique that supports motor recovery in patients after stroke [50]. However, despite the growing interest in FPS, there is a lack of high-quality research on its effectiveness in neurological rehabilitation programmes [13].

This interventional, controlled, randomised, double-blinded trial assessed the effects of rehabilitation via functional proprioceptive stimulation on balance and functional efficiency in patients after stroke. After 4 weeks, patients in both the VG and SG groups had achieved significant improvements in most of the studied parameters. However, the VG group showed significantly greater improvement in balance and functional efficiency the SG group, as demonstrated by the following tests: BBS, FRT and BI, as well as codes from the ICF rehabilitation set: D420 and D640. Furthermore, the improvement in BBS and BI scores in the VG group exceeded the threshold for what is considered a minimally clinically important change [51, 52].

The results of this study revealed a significantly greater improvement in functional balance in the VG group than in the SG group. Similar results were obtained by Karimi-Ahmad Abadi et al., who showed that conventional physiotherapy combined with vibration increased the change in the median BBS score by 3 points in the vibration group, whereas no effects were observed in the control group. The good results in improving postural stability in people after stroke may be due to the fact that vibrations activate and increase the activity of the somatosensory cortex [53]. Therefore, muscle-tendon vibration can reduce the loss of sensation and influence the stimulation of sensation on the paretic side, which can lead to increased symmetry in the loading of the lower limbs [54]. However, according to the review by Alashram et al., high-quality evidence on the effect of focal muscle vibration is still limited [55].

In neurorehabilitation, rehabilitation protocols aim to restore impaired motor skills, which can be achieved by performing physical exercises combined with proprioceptive training [56]. These changes could be attributed to a better agonist/antagonist interplay because of a rearrangement in central and segmental nervous pathways. Consequently, the power and absolute force are increased, the kinematics are smoother, and the articular efficiency is greater [28]. The main mechanism by which FPS can enhance motor recovery is through direct effects on the motor cortex. Repetitive vibrations stimulate and generate sensory inputs that reach the cortical areas and improve function through.

a mechanism related to the phenomenon of neuropathy [57]. To ensure effective and lasting motor regeneration, stimulation of the sensorimotor system should be implemented at an early stage of rehabilitation.

FPS training in a standing position aimed at improving postural control combined with conventional rehabilitation significantly improved the functional status of the patients studied. The change observed was significantly greater in the VG group than in the SG group. Toscano et al. reported that repetitive focal muscle vibration can improve motor function in patients after stroke. The authors reported a significant improvement in motor indices in the FPS group compared with the control group [15].

The maximum recovery of function and independence after stroke is a priority in medical treatment. Motor rehabilitation should be based on regular assessments of motor function and activity by adopting the comprehensive ICF framework to evaluate the patient’s functional status. This study found that patients in the VG group showed significant improvements in D420—Transferring oneself and D640—doing housework activities, compared with those in the SG group. Task-oriented training aims to improve motor function and is based on the principle that repeated exercises/stimulations are the best way to learn a specific task [58]. Transferring is a fundamental skill that is essential for independent movement and increased activity in stroke survivors [59]. The results of our own studies confirm the effectiveness of supplementing conventional therapy with FPS therapy in patients after stroke. This combination resulted in faster and larger improvements in balance and functional efficiency in people after a stroke. Due to its non-invasive nature, safety and limited number of contraindications, this technology has the potential to become an important complementary non-pharmacological method in physiotherapy, supporting patient’s recovery of functional efficiency after stroke.

### Limitations

This study has several limitations. Firstly, no follow-up assessment was planned after the intervention was completed to check how long the rehabilitation effect would last. Due to the specific nature of the rehabilitation centre and the reduction in care for stroke patients from across Poland, it was not possible to perform another measurement. The population of stroke patients is very diverse. The inclusion criteria adopted in the study prevented the assessment of the effectiveness of the intervention supplemented with FPS in all stroke patients, regardless of their functional status and the period after stroke. This may affect the generalisability of the results.

## Conclusions

Early and intensive rehabilitation is essential for stroke recovery. However, the literature lacks clear consensus on the most effective protocol for improving patient outcomes. This study aimed to evaluate the impact of rehabilitation incorporating functional proprioceptive stimulation on postural control and functional performance in poststroke patients. Results showed that both conventional rehabilitation and rehabilitation with FPS improved balance and functional efficiency. Notably, the FPS group demonstrated significantly quicker and greater gains than the placebo group, with clinically meaningful improvements in BBS and BI scores. These findings suggest that combining conventional therapy with repetitive focal muscle vibration may enhance recovery. Further research is needed to assess the long-term effects of integrating FPS into stroke rehabilitation.

## Data Availability

The datasets generated and analysed during the current study are available in the University of Rzeszow repository, https://repozytorium.ur.edu.pl/items/1c2b018f-0926-4337-bb42-218dc3edb3ff.

## Abbreviations

FPS: Functional Proprioceptive Stimulation
RCT: randomized controlled trial
BBS: Berg Balance Scale
FRT: Functional Reach Test
BI: Barthel Index
TIS: Trunk Impairment Scale
PL: Center of gravity
S: Area
XD: Deviation in the X axis
YD: Deviation in the X axis
ASX: Average velocity in the X axis
ASY: Average velocity in the Y axis
ICF: The International Classification of Functioning, Disability and Health
